# Temperature to time Catch-Up: a novel procedural endpoint to predict durable pulmonary vein isolation after cryoballoon ablation of paroxysmal atrial fibrillation

**DOI:** 10.1007/s00392-023-02361-7

**Published:** 2023-12-19

**Authors:** Kevin Willy, Julian Wolfes, Patrick Müller, Christian Ellermann, Dirk Dechering, Philipp S. Lange, Benjamin Rath, Florian Reinke, Florian Doldi, Fatih Güner, Julia Köbe, Patrick Leitz, Gerrit Frommeyer, Mikael Laredo, Lars Eckardt

**Affiliations:** 1 Department of Cardiology II-Electrophysiology, University Hospital of Münster, Albert-Schweitzer-Campus 1, 48419 Münster, Germany; 2 Department of Electrophysiology, Klinikum Vest Recklinghausen, Recklinghausen, Germany; 3 Department of Cardiology, Niels-Stensen-Kliniken Marienhospital Osnabrück, Osnabrück, Germany; 4 Department of Cardiology and Electrophysiology, Hôpital Pitié-Salpêtrière, Paris, France

**Keywords:** Cryoballoon ablation, Atrial fibrillation, Ablation parameters, Pulmonary vein isolation, Recurrence

## Abstract

**Background:**

Cryoballoon ablation is a widely used single-shot technique for pulmonary vein isolation (PVI) in the treatment of paroxysmal atrial fibrillation (AF). Procedural endpoints ensuring maximal PVI durability are important.

**Objective:**

To assess the performance of cryoablation procedural markers to predict long-term PVI.

**Methods:**

In a single center, consecutive patients who underwent redo ablation with high-density mapping for symptomatic AF recurrence after cryoballoon ablation were included and cryoballoon procedural data were collected, including temperature values at 30 and 60 s, time to isolation, nadir temperature and the velocity of temperature decline estimated with the temperature/time catch-up point (T2T-Catch-Up) defined as positive when the freeze temperature in minus degree equals the time in seconds after cryoablation initiation (e.g. − 15 °C in the first 15 s of the ablation impulse).

**Results:**

47 patients (62% male; 58.3 ± 11.2 years) were included. Overall, 38 (80.9%) patients had ≥ 1 reconnected PV. Among 186 PVs, 56 (30.1%; 1.2 per patient on average) were reconnected. Univariate analysis revealed T2T-Catch-Up in 103 (56%) and more frequent in durably isolated than in reconnected PVs (93 [72%] vs 10 [19%], *p* < 0.0001). Among binary endpoints, T2T-Catch-Up had the highest specificity (82%) and predictive value for durable PVI at redo ablation (90%). In multivariable analyses, absence of T2T-Catch-Up (Odds-ratio 0.12, 95% CI [0.05–0.31], *p* < 0.0001) and right superior PV (Odds-ratio 3.14, 95% CI [1.27–7.74], *p* = 0.01) were the only variables independently associated with PV reconnection.

**Conclusion:**

T2T-Catch-Up, a new and simple cryoballoon procedural endpoint demonstrated excellent predictive value and strong statistical association with durable PVI.

**Graphical abstract:**

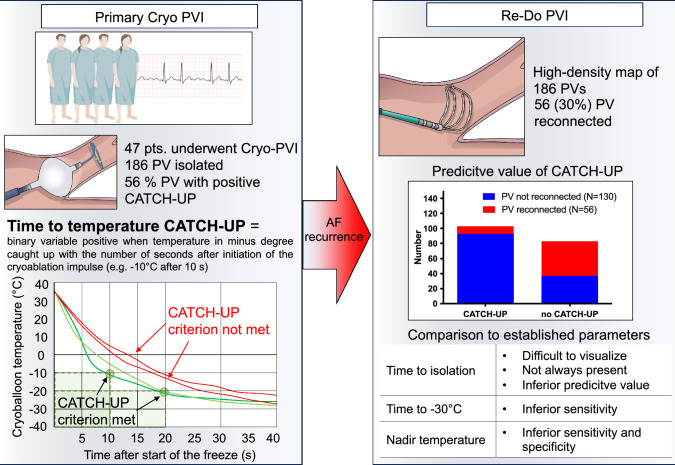

## Introduction

Cryoballon ablation for atrial fibrillation (AF) has evolved to a reasonable first-line therapy [1] and recent data have underlined the superior outcome of early cryoablation over AAD [2]. However, AF recurrences are common and mostly caused by pulmonary vein (PV) reconnections. Previously published data of the FIRE AND ICE trial indicated the superiority of cryoablation over RF ablation, but still 1.4 PV reconnections per patient during repeat procedures after cryoballon ablation were observed [3].

Procedural markers of lesion quality are important to guide cryoballon ablation and to predict procedural success. In particular, with the refinement of pulmonary vein isolation (PVI) energy and constant increase in durable PVI rate, procedural markers with a high predictive value for durable PVI are needed.

Temperature at 30 s, at 60 s, thaw time and time to isolation (TTI) are independently associated with PVI durability after cryoballoon ablation [4]. However, visualization of TTI is not always possible and these parameters may only partially reflect the quality of the initial phase of freezing. We, therefore, assessed parameters trying to predict durable PVI in a setting of optimal isolation control by performing a second PVI procedure with HD mapping after an initial cryoballoon ablation of AF and systematically analyzing data from the initial Cryo PVI. Here, in addition to the assessment of already established parameters, we also aimed at identifying potentially new parameters to improve prediction for PV reconnection after a cryoballoon procedure. We hypothesized that a parameter that may express the velocity of the temperature decline throughout the freeze while being independent of the PV signal visualization could be a marker for PV occlusion. (I) Especially a parameter reflecting the early phase of the freeze as probably one of the most decisive parts in terms of complete occlusion and contact with the PV wall might be of interest. (II) Furthermore, the parameter should ideally be independent of other requirements such as PV signal visualization not being acquired in all cryoablation freezes. Finally (III), a binary parameter would be of particular value to define thresholds that may aid the continuation or abortion of a cryofreeze.

## Methods

### Study cohort

The study cohort consisted of consecutive patients undergoing a repeat procedure with a three-dimensional high-density mapping guided radiofrequency ablation for recurrent atrial arrhythmias from 01/2019 to 04/2020 (time of the performance of the redo procedures) after a first-time cryoballoon ablation (time of initial ablation: 04/2016–08/2019) for the treatment of symptomatic AF. Only patients with complete procedural and biophysical datasets of both ablation procedures were included in the study. All procedures were performed at the University Hospital of Münster, Münster, Germany.

### Index cryoballoon ablation

The cryoablation was performed as described before [5]. In short, diagnostic catheters were positioned in the coronary sinus and in the right ventricular apex. Subsequently, a single transseptal puncture was performed under fluoroscopic guidance. Angiograms of the pulmonary veins were performed with a multipurpose catheter (MP1SH, Boston Scientific Inc., Natick, MA, USA). Thereafter, a 28-mm cryoballoon catheter (ArcticFront Advance, Medtronic, Minneapolis, MN, USA) was introduced into the left atrium. The cryoballoon was placed at each PV ostium guided by the achieve-mapping-catheter. Heparin was administered adjusted to body weight before transseptal puncture and thereafter ACT-guided. In patients in AF, electrical cardioversion was performed before ablation to facilitate PV potentials discrimination. In the right superior and inferior veins, freezes were delivered under continuous stimulation of the phrenic nerve. Freezes were aborted if phrenic palsy occurred, or the temperature fell below − 65 °C. The number of ablation freezes ranged from 1 to 4 while in most cases one freeze of 180 s or 240 s depending on the achievement of TTI was delivered. Complete PV isolation was confirmed by entrance/exit block using the achieve-mapping catheter and in case of difficult discrimination by adenosine injection. If AF was present at the end of the procedure, electrical cardioversion was performed again.

### In-hospital management

PVI was performed in accordance with international guidelines and clinical standards with a continuation of anticoagulation during ablation and previous transesophageal echocardiography to exclude atrial thrombus and facilitate transseptal puncture routinely performed in all cases.

### Redo-PVI procedure

All patients scheduled for Redo-PVI had symptomatic documented AF recurrence after Cryo-PVI. All Redo-PVI procedures were performed using 3D high-density mapping (NavX, St. Jude Medical, St Paul, Minnesota). PV reconnection was defined as the presence of sharp near-field PV potential within the PV associated with atrial capture upon PV pacing. If PV reconnection could be proven, RF ablation was performed until complete isolation was proven by re-map as well as loss of PVP. Isolation was controlled by stimulation from the left atrium as well as the coronary sinus and the right ventricle in case of difficult discrimination of the ventricular signal.

### Patient and procedural data

Data on patients’ medical history, medication and demographics was taken from medical records. Procedural data was obtained from the ablation protocols, the ablation report and data obtained from the console of the cryoballoon ablation system (Medtronic), in which the temperature course of any freeze delivered is stored. In addition to basic data such as the number and time of freezes delivered, we extracted further parameters such as the temperature at 30 and 60 s of the ablation impulse, nadir temperature, time-to-isolation (TTI) and time-to-temperature (TTT). Furthermore, we defined an additional parameter, the presence of temperature-to-time-catch-up, referred to as T2T-Catch-Up, a binary variable positive when temperature in minus degree caught up with the number of seconds after initiation of the cryoablation impulse (central illustration). The corresponding T2T-Catch-Up time, a continuous variable, was calculated in PVs with T2T-Catch-Up, i.e. 12 s for T2T-Catch-Up at − 12 °C after 12 s duration of the respective freeze. Thaw time was recorded, defined as the time the PV required to warm up from − 30 to + 15 °C according to the literature [4, 6]. In case of two freezes in the same vein, the longer one or, if both were equally long, the first one, was included in the analysis.

### Statistical analyses

Continuous variables were expressed as mean ± SD or median [interquartile range, IQR] for normally and non-normally distributed data, respectively, and were compared using Student’s *t* test or the Mann–Whitney–Wilcoxon test, as appropriate. Categorical variables were expressed as the frequency (percentage) and compared using a chi-squared test or Fisher’s exact test. Diagnostic performances of cryoballoon procedures characteristics to predict PVI durability were assessed by sensitivity (SEN), specificity (SPE), negative predictive value (NPV) and positive predictive value (PPV) for binary variables and area under the receiver operating characteristic (ROC) curve (AUC) for continuous variables. Cutoffs among continuous variables were selected using the highest Youden index on the ROC curve and according to commonly used values in the literature. Regression analyses involved nominal logistic regression. Variables with *p* < 0.10 in univariate analyses were included in a multivariate model.

All tests were two-tailed, and the threshold for statistical significance was set to *p* < 0.05. Statistical analyses were performed with JMP v15.2.0 (SAS Institute Inc., Cary, NC) and schematics were created by GraphPad Prism v9.3.0 (GraphPad Software, San Diego, CA).

## Results

### Index cryoablation

Characteristics of patients at the time of the cryoablation procedure are shown in Table 1. Most patients had paroxysmal AF (30, 63.8%) and the symptom burden was high with a median EHRA class of III. Median procedural time was 85 (75–100) min and fluoroscopic time 13.5 (11.5–17.4) min. All patients were in sinus rhythm at the end of the procedure and at hospital discharge.

**Table 1:** Patient characteristics at cryoablation procedure (*n* = 47)

Male sex	29 (61.7%)
Age at cryoablation (years)	60 (53–65)
BMI (kg/m^2^)	27.1 ± 3.6
Comorbidities	
Hypertension	25 (53.2%)
Diabetes mellitus	3 (6.4%)
Coronary artery disease	3 (6.4%)
Congestive heart failure	2 (4.3%)
History of stroke/TIA	3 (6.4%)
Paroxysmal AF	30 (63.8%)
CHA_2_DS_2_-Vasc-Score	1 (1–2)
EHRA class	3 (3–4)
*N*. of failed anti-arrhythmic drugs	1 (1–1)
LVEF (%)	59.5 (54–61.3)
Index left atrial volume (ml/m^2^)	37 (30–45)

Data are *n* (%), median (interquartile range) or mean ± SD

*LVEF *left ventricular ejection fraction, *EHRA *European Heart Rhythm Association

### Redo PVI

Redo procedures were performed 367 ± 244 days after the index cryoablation procedure. There were 56 PV reconnections (30.1% of all 186 PVs) in 38 (80.9%) patients. PV reconnection occurred in 20 right superior (35.7%), 14 right inferior (25%), 9 left inferior (16) and 13 left superior (23%) PVs. Temperature at 30 s, 60 s, and nadir temperature of the initial application was significantly lower in durably isolated PVs, whereas thaw time was significantly longer (Table 2). PVI was visualized in 73 (39%) PVs overall and was significantly more visualized in durably isolated PVs than in reconnected PVs; among 73 PVs with time to isolation (TTI) available, TTI was not significantly different between durably isolated PVs and reconnected PVs. T2T-Catch-Up was present in 103 (56%) PVs. T2T-Catch-Up time was 22 s (14–27) on average.

**Table 2:** Cryoballoon procedure characteristics according to PV reconnection status at redo-PVI

	All PVs (*n* = 186)	Durably isolated PVs (*n* = 130)	Reconnected PVs (*n* = 56)	*p *value
Number of freezes, *n*	1 (1–2)	1 (1–2)	1 (1–2)	0.84
Freeze duration, s	180 (180–323)	180 (180–300)	180 (180–360)	0.58
Temperature at 30 s, °C	− 28 (− 32 to − 22°)	− 29 (− 34 to − 24°)	− 22.5 (− 28 to − 19°)	< 0.0001
Temperature at 30 s ≤ − 30 °C	74 (40)	64 (49)	10 (18)	< 0.0001
Temperature at 30 s ≤ − 23 °C	137 (74)	109 (84)	28 (50)	< 0.0001
Temperature at 60 s, °C	− 79 (− 34 to − 42°)	− 39 (− 44 to − 35°)	− 35 (− 38 to − 31°)	< 0.0001
Nadir temperature	− 45 (− 50 to − 40°)	− 47 (− 52 to − 38°)	− 42 (− 45 to − 37°)	< 0.0001
Thaw time, s	27 (19–37)	29 (23–38)	20 (17–28)	< 0.0001
CATCH-UP	103 (56)	93 (72)	10 (18)	< 0.0001
CATCH-UP time, s^a^	22 (14–27)	21 (13–27)	24.5 (19–28)	0.15
Visualization of PV isolation	73 (39)	60 (46)	13 (23)	0.003
Time to isolation, s^b^	30 (25–50)	30 (25–50)	45 (25–75)	0.24
Time to isolation < 60 s^b^	59 (86)	49 (86)	10 (83)	0.82

Data are *n* (%), median (interquartile range) or mean ± SD

*CATCH-UP* presence of time-to-temperature catch-up, *CATCH-UP time* time to time-to-temperature-catch-up, *PV* pulmonary vein, *PVI* pulmonary vein isolation

^a^Among 103 PVs with CATCH-UP

^b^Among 73 PVs with visualization of PV isolation

### Diagnostic performances

Among continuous variables, temperature at 30 s and nadir temperature had the higher ROC AUCs (0.73 and 0.72, respectively) (Table 3).

**Table 3:** Diagnostic performances of cryoablation procedure characteristics for durable *PV* isolation

Continuous variables	ROC AUC (95% CI)
Temperature at 30 s, °C	0.73 (0.65–0.80)
Temperature at 60 s, °C	0.69 (0.61–0.78)
Nadir temperature, s	0.72 (0.64–0.80)
Thaw time, s	0.69 (0.61–0.77)
Time to isolation (TTI)	0.60 (0.43–0.78)
CATCH-UP time	0.64 (0.50–0.78)

Binary variables	Se, % (95% CI)	Spe, % (95% CI)	PPV, % (95% CI)	NPV, % (95% CI)
Temperature at 30 s < − 30 °C	49 (40–58)	82 (70–91)	86 (77–93)	41 (32–51)
Temperature at 30 s < − 23 °C	84 (77–89)	50 (37–63)	80 (77–89)	57 (43–70)
Temperature at 60 s < − 40 °C	46 (37–55)	80 (67–90)	85 (74–92)	39 (30–48)
Nadir temperature < − 45 °C	64 (55–72)	73 (60–84)	85 (76–91)	47 (9–24)
CATCH-UP	72 (63–79)	82 (83–95)	90 (83–95)	55 (44–66)
Visualization of PV isolation	46 (37–55)	77 (64–87)	82 (71–90)	38 (29–47)

*AUC* area under the curve, *CATCH-UP* presence of time-to-temperature catch-up, *CATCH-UP time* time to time-to-temperature-catch-up, *NPV* negative predictive value, *PPV* positive predictive value, *PV* pulmonary vein, *PVI* pulmonary vein isolation, *ROC* receiver operating characteristics, *Se* sensitivity, *Spe* specificity

**Fig. 1:**
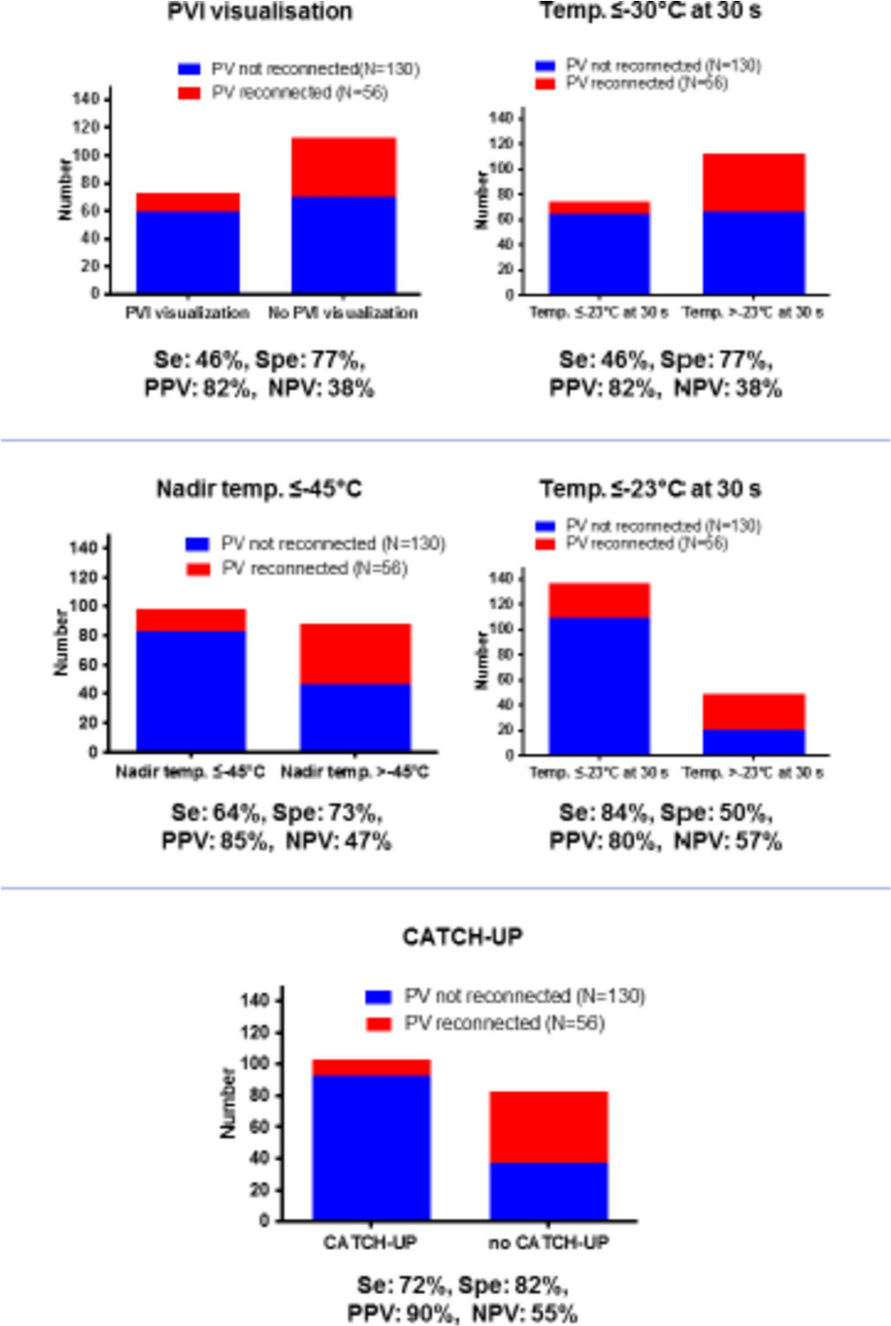
Diagnostic yield of different parameters for the prediction of durable PVI

A temperature of 30 s of 23 °C and a nadir temperature of − 45 °C had the higher Youden index and were selected as binary variables. T2T-Catch-Up time and TTI had lower AUCs (0.64 and 0.60, respectively), but were calculated in a smaller sample. Among binary variables (Fig. 1), T2T-Catch-Up had the highest specificity (82%) and PPV (90%). Temperature at 30 s ≤ − 30 °C had a similar specificity (82%) but lower sensitivity (49% vs 72%). Temperature at 30 s ≤ − 23 °C, the cutoff value with the highest Youden index, had a higher sensitivity than T2T-Catch-Up (84% vs 72%) but lower specificity (50% vs 82%), lower PPV for durable PVI (80% vs 90%) and diagnostic accuracy overall.

### Regression analyses

In univariate analyses, temperature at 30 s, temperature at 60 s, nadir temperature, thaw time, T2T-Catch-Up, PVI visualization and right superior PV were all significantly associated with durable PVI (Table 4). In multivariate analysis, the absence of T2T-Catch-Up (Odds-ratio 0.12, 95% CI [0.05–0.31], *p* < 0.0001) and the right superior PV (Odds-ratio 3.14, 95% CI [1.27–7.74], *p* = 0.01) were the only variables independently associated with PV reconnection.

**Table 4:** Logistic regression analyses to assess the association between PV isolation durability and cryoballoon procedural characteristics

	Univariable			Multivariable		
	Odds-ratio	95% CI	*p *value	Odds-ratio	95% CI	*p* value
Temperature at 30 s, °C	1.09	1.04–1.14	< 0.0001	0.99	0.94–1.05	0.86
Temperature at 60 s, °C	1.10	1.04–1.16	< 0.0001	0.99	0.93–1.05	0.68
Nadir temperature, s	1.02	1.07–1.20	< 0.0001	1.02	0.93–1.12	0.65
Thaw time, s	0.94	0.91–0.97	< 0.0001	0.98	0.93–1.03	0.98
CATCH-UP	0.09	0.04–0.18	< 0.0001	0.12	0.05–0.31	< 0.0001
Visualization of PV isolation	0.35	0.17–1.39	0.004	0.70	0.30–1.60	0.39
RSPV	2.12	1.06–4.23	0.03	3.14	1.27–7.74	0.01

*CATCH-UP* presence of time-to-temperature catch-up, *CATCH-UP time* time to time-to-temperature-catch-up, *NPV* negative predictive value, *PPV* positive predictive value, *PV* pulmonary vein, *PVI* pulmonary vein isolation, *Se* sensitivity, *Spe* specificity

## Discussion

In a retrospective study assessing the diagnostic performance of cryoballoon procedural characteristics in 186 PVs (47 patients) with Redo AF ablation and 3D high-density mapping, we systematically evaluated cryo procedure data of a first cryoballoon ablation with regard to long-term PV isolation after symptomatic AF recurrence. Our study showed that a new procedural marker, the presence of temperature-to-time catch-up, available for every PV freeze and independent of PVI visualization, had the highest predictive value for durable PVI and outperformed established parameters such as TTI or temperature values at fixed time points during the freeze.

As compared to TTI, T2T-Catch-Up was observed in 56% of PVs versus 39%, had higher specificity and sensitivity and consequently higher predictive values for durable PVI. T2T-Catch-Up was the only procedural endpoint independently associated with durable PV in multivariable analysis and had the strongest association with durable PVI (OR for PV reconnection 0.12). In addition, T2T-Catch-Up had the best predictive value for durable PVI but the absence of T2T-Catch-Up did not predict PV reconnection well. This was also the case for all procedural markers assessed with a maximal predictive value for PV reconnection given by temperature at 30 s > − 23 °C of only 57%, despite a sensitivity of 84%. This might statistically be explained partly by the low prevalence of reconnected PVs. In other words, if T2T-Catch-Up is reached, durable PVI is almost guaranteed. But if T2T-Catch-Up is not reached, reconnection is not necessarily very likely. To ensure the maximal probability of durable PVI, T2T-Catch-Up-based strategy seems a reasonable target of Cryo PVI ablation. All Cryo-PVIs as well as the redo procedures were done following the same protocol, same catheter set-up and mapping system. Furthermore, all parameters were prospectively collected thoroughly. All trials of drug treatment of AF in the meantime were documented as well as clinical symptom burden. However, the number of patients in this clinical constellation was small, so larger trials are warranted to examine to usefulness of T2T-Catch-Up in the prediction of successful long-term PVI. In previous studies, a TTI < 60 s was the best predictor of durable PV isolation [6]. However, the freeze duration was much longer than in current ablation protocols. TTI-guided cryoenergy titration reduced procedure duration and fluoroscopy time but not long-term freedom from atrial arrhythmias [7]. In this study, T2T-Catch-Up could not be compared directly to TTI because of a lack of quantitative data among the latter but outperformed visualization of PV isolation in both sensitivity and specificity. T2T-Catch-Up was valuable for both, patients with paroxysmal as well as persistent AF, making it useful for a large population of patients scheduled for PVI. The results may also provide new insights into a “perfect” cryofreeze and how to measure it with surrogate parameters. Ghosh et al. demonstrated that an arrest in temperature rise might correlate with melting the tissue ice formed near the balloon so that a long warming duration may indicate a thicker ice layer and a more durable isolation. Besides, ice crystal formation with longer warming times may also be associated with additional tissue damage by cryoenergy [4, 8]. In contrast, our results indicate the importance of the freezing component of the ablation impulse rather than the thawing time. These findings are supported by a recent study by Miyazaki et al. [9] who showed, that a faster freezing speed was most important for the prediction of a durable PVI in the LSPV, the LIPV, and the RSPV whereas a slower thawing speed was only superior for RIPV isolation prediction. Our data support the hypothesis that fast cooling and slow thawing are most efficacious in creating a durable PV lesion. One could hypothesize that this is an expression of optimal tissue contact between catheter/balloon and PV wall contact and catheter stability, which have extensively been proven as good markers of lesion quality in RF ablation procedures [10, 11].

T2T-Catch-Up might serve as a valuable novel marker for successful PV isolation.

In this study, we did not investigate whether a certain time of T2T-Catch-Up might serve as a cutoff value beyond the sole presence of T2T-Catch-Up; as the study sample was too small for reliable evaluation. A larger multicentric evaluation is ongoing as a result of this study to assess T2T-Catch-Up time as a quantitative variable and compare it with TTI and also assess the reproducibility of T2T-Catch-Up predictive values across different centers with different cryoballoon setups. In addition, future trials should evaluate its use as a primary endpoint in ablation protocols and may investigate freeze abortion if T2T-Catch-Up is not reached. Until now, TTI is seen as the “gold standard” of procedural outcome to optimize the chance for durable PVI. However, TTI-guided cryoenergy titration reduced procedure duration and fluoroscopy time but not long-term freedom from atrial arrhythmias [7]. Therefore, the use of T2T-Catch-Up as a new marker might be of additional clinical value. Future prospective trials may also analyse ablation success and lesion formation aided by imaging modalities such as cardiac MRI to evaluate how achievement of T2T-Catch-Up influences lesion size and depth but also collateral damage to the surrounding tissue as lesion analysis with cardiac MRI has also been shown promising results regarding lesion quality and durability [11–13].

### Study limitations

First, this is an observational study that lacks randomization. In addition, we recorded PV isolation (and thus TTI) in only 39% of PVs, so that TTI as a continuous variable was difficult to assess. Therefore, in future trials to assess the T2T as a predictive parameter of PV ablation success, the index cryo procedure should indeed be designed for optimal visualization of PVP and PVI success to further evaluate the positive evidence gained in our study. The addition of a control group without AF recurrence after cryoablation from our centre was omitted as no structured follow-up program existed and most patients without recurrence were not re-admitted to our centre so data quality would not have been sufficient for such a study design. Secondly, all cryoballoon procedures were performed with a single manufacturer’s device and the results may not apply to other comparable devices. Third, the relatively small sample size required PV-specific rather than patient-specific analysis, which has to be taken into account interpreting the results. A recent study showed a significant discrepancy in the ability of TTI to predict durable PVI across PVs [14]. Last, larger studies with clinical endpoints are warranted to assess the clinical utility of T2T-Catch-Up.

## Conclusions

T2T-Catch-Up, a novel cryoballoon procedural marker available for every application, demonstrated excellent predictive value and strong association with durable PVI, outperforming contemporarily used parameters such as TTI or temperature values at fixed time points of the cryo impulse in our study. Future multicenter studies need to define T2T-Catch-Up quantitative thresholds and assess their value to predict clinical endpoints and further guide the way for a possible ideal cryoballoon freeze to deliver.

## Data Availability

Raw data is available upon reasonable request from the corresponding author.
